# Case Report: Heart Transplantation for Refractory *Candida tropicalis* Endocarditis: A Case Report and Literature Review

**DOI:** 10.3390/microorganisms13030580

**Published:** 2025-03-04

**Authors:** Ricardo El Nouwar, Eva Larranaga Lapique, Frédéric Vanden Eynden, Delphine Martiny, Myriam Remmelink, Ana Roussoulières, Maya Hites

**Affiliations:** 1 Clinic of Infectious Diseases, Hôpital Universitaire de Bruxelles (HUB), 1070 Brussels, Belgium; 2 Department of Cardiac Surgery, Hôpital Universitaire de Bruxelles (HUB), 1070 Brussels, Belgium; 3 Laboratoire Hospitalier Universitaire de Bruxelles-Université Libre de Bruxelles (LHUB-ULB), 1000 Brussels, Belgium; 4 Anatomy and Pathology Department, Hôpital Universitaire de Bruxelles (HUB), 1070 Brussels, Belgium; 5 Department of Cardiology, Hôpital Universitaire de Bruxelles (HUB), 1070 Brussels, Belgium

**Keywords:** fungal endocarditis, heart transplantation, antifungal therapy, *Candida* infection

## Abstract

*Candida* infective endocarditis presents therapeutic challenges with high mortality. A complex case of *Candida* prosthetic valve endocarditis refractory to standard antifungals (anidulafungin and fluconazole) and high-dose caspofungin was successfully treated with heart transplantation. The literature review revealed a few cases of bacterial endocarditis successfully treated with heart transplantation, but with only two transplanted cases of fungal endocarditis. This report explores heart transplantation as a last resort for managing refractory infective endocarditis. The patient is still alive and free of infection, two and a half years after transplantation.

## 1. Introduction

*Candida* infective endocarditis (IE) remains a rare entity, with scarce literary evidence to orient its treatment. Causative agents are evenly distributed between all *candida* species, although more recent findings suggest an increase in the non-albicans group. *Candida* species, including *Candida tropicalis*, exhibit virulence factors such as biofilm formation, adhesins, secretion of phospholipases and proteinases, phenotypic switching, and immune evasion through interference with phagocytosis which contribute to its pathogenicity in cardiovascular infections [1]. Current guidelines are based on expert opinions rather than clinical trials. The current recommendation regarding *Candida* IE is to associate surgery with an antifungal agent like amphotericin B, followed by a lifelong suppressive oral antifungal agent, due to a high risk of relapse. Even with treatment, mortality rates remain unacceptably high, exceeding 50% [2].

## 2. Case Presentation

**Figure 1:**
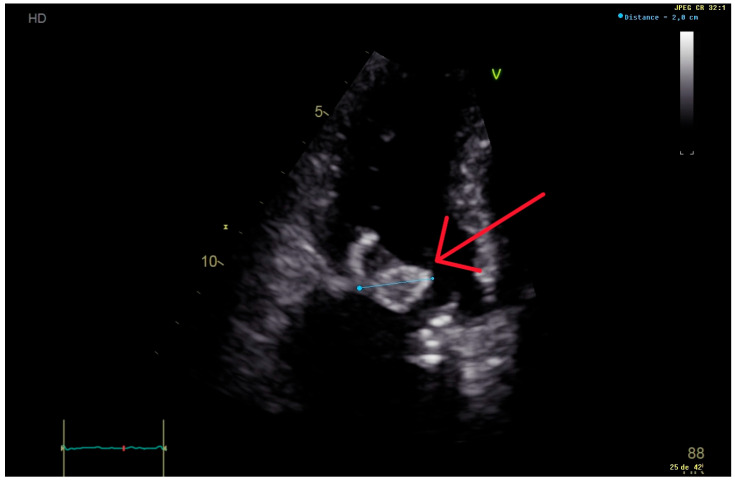
Trans-thoracic echocardiography performed 6 months after the first valvular replacement, showing a 20 mm vegetation on the mitral prosthetic valve (red arrow), causing severe intraprosthetic obstruction.

**Figure 2:**
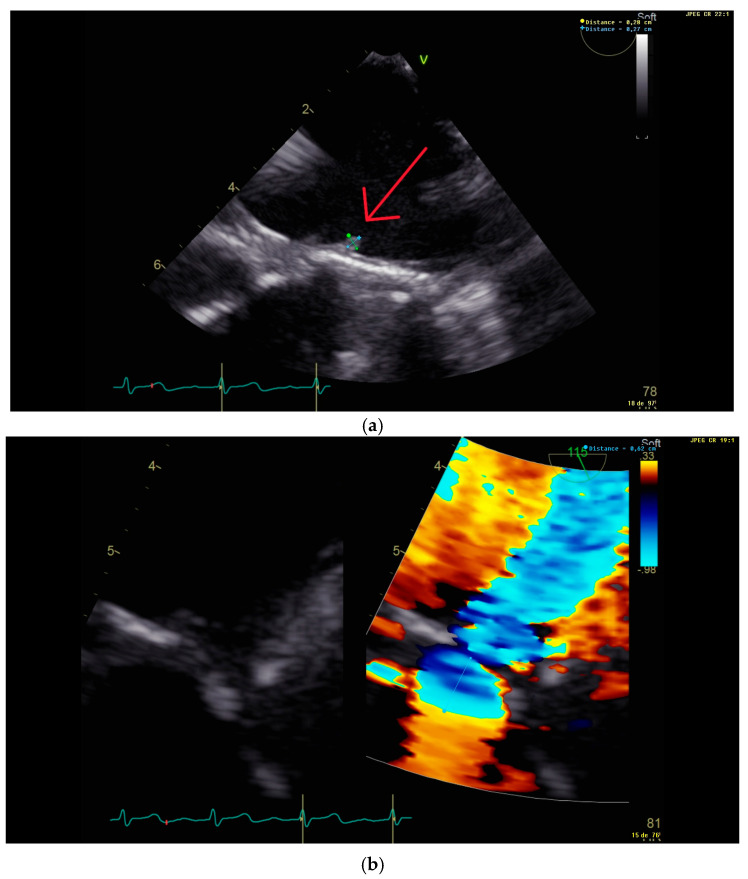
Trans-esophageal echocardiography performed 2 months after the second valvular replacement, showing (**a**) a 3 mm vegetation (red arrow) on the new prosthetic mitral valve (below) and (**b**) mitral insufficiency with a proximal isovelocity surface area of 6 mm.

**Figure 3:**
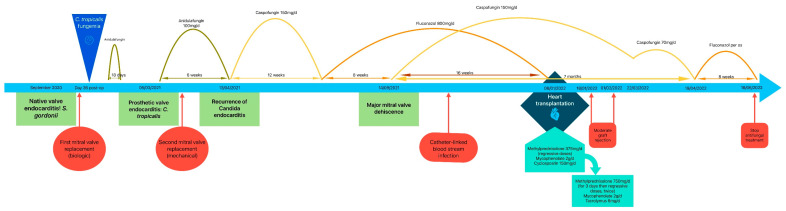
Timetable showing the different main events and treatments leading to heart transplantation, with follow up.

We present a complicated case of fungal endocarditis in a 63-year-old male patient admitted for palpitations and dyspnea. Six months prior to presentation, the patient underwent a mitral biological valve replacement in another hospital for decompensated heart failure due to a native mitral valve *Streptococcus gordonii* endocarditis. His past medical history revealed cases of hemochromatosis, arterial hypertension, hypercholesterolemia, malignant melanoma, and chronic kidney disease with a glomerular filtration rate of 56 mL/min/1.73 m^2^ according to the 2021 Chronic Kidney Disease Epidemiology Collaboration equation. He had no known allergies. His mitral valve replacement was complicated on day 36 post-operation by fungemia due to *Candida tropicalis*, which was treated by anidulafungin for 18 days (first documented negative culture on D8 of treatment, no blood cultures performed before 8 days of treatment). Upon admission 6 months after his mitral valve replacement, his blood cultures tested positive over five consecutive days for *Candida tropicalis* with the same previous resistance profile according to the Clinical and Laboratory Standards Institute guidelines of 2021 (fluconazole MIC = 1 μg/mL susceptible, anidulafungin MIC = 0.12 μg/mL susceptible, caspofungin MIC = 0.06 μg/mL susceptible) [3]. Trans-thoracic echocardiography showed a 20 mm vegetation on the mitral prosthetic valve with the thickening of its leaflets (Figure 1). Anidulafungin 100 mg per day was initiated (no loading dose given). Further workup showed a spondylodiscitis L2–L3 extending one month later to Th12-L1 on a positron emission tomography scan (PET-scan), with suspicions of septic emboli due to the presence of hypermetabolic lesions on the seventh costal rib and bilateral hilar lymph nodes. A small ischemic embolic cerebral lesion was also documented in the right occipital lobe via magnetic resonance imaging (MRI). Surgical replacement of the prosthetic biological valve with a mechanical one was performed on D5 of anidulafungin; surgical samples were culture-positive for *Candida tropicalis*. After six weeks of Anidulafungin, the patient still had night sweats. A new trans-esophageal echocardiography showed a new 3 mm vegetation with mitral valve insufficiency (Figure 2). His CRP increased from 21 to 74 mg/L. A new MRI of the spine was performed, showing no signs of improvement of his spondylodiscitis. The patient was readmitted, and therapy was changed. Combination therapy with amphotericin B was not given due to his decreased kidney function. Anidulafungin was switched to caspofungin 150 mg/day (high-dose) for a body weight of 74 kg, followed by positive clinical, biological, and radiological responses (Figure 3). After 3 months of high-dose caspofungin, treatment was shifted to high oral dose fluconazole (800 mg per day). Eight weeks after the switch, a follow-up echocardiography showed significant mitral valve dehiscence. The patient reported progressive dyspnea evolving to heart failure, requiring hospitalizations for acute decompensated heart failure. New blood cultures and a discitis biopsy remained negative. Caspofungin (150 mg per day) was reintroduced. A PET-scan, repeated three months after initiation of dual antifungal therapy, showed the resolution of the cardiac metabolic abnormalities, regression of the spondylodiscitis lesions, and observed regression of metabolic activity in the bilateral mediastino-hilar and hepatic peri-hilar lymph nodes. Faced with mitral valve dehiscence, and with no other surgical options in a patient with a controlled disseminated fungal infection, our multidisciplinary team decided to list the patient for a heart transplantation. Concomitantly to this decision, the new PET-scan revealed new diffuse and heterogenous hepatic hypermetabolism, which is suggestive of drug-induced hepatitis. However, liver echography remained normal.

Heart transplantation was performed 15 weeks after listing the patient in Eurotransplant. Immunosuppressive treatment (IS) regimen differed from the one usually administered in our hospital; no induction treatment by thymoglobuline or basiliximab was administered. Initially, intravenous, followed by oral, methylprednisolone was administered in association with cyclosporin (instead of tacrolimus) and mycophenolate mofetil as IS maintenance treatment. Three days before transplantation, caspofungin dosage was lowered to 70 mg/day and fluconazole was stopped due to probable hepatotoxicity (alkaline phosphatase 634 U/L, gamma-glutamyl transferase 1407 U/L, alanine aminotransferase 279 U/L, aspartate aminotransferase 140 U/L). His liver enzymes improved over the following days and caspofungin dosage increased back to 150 mg three days post-transplantation. Caspofungin drug monitoring was performed while being administered 150 mg per day, with measured serum concentrations of 7.615 µg/mL and 7.678 µg/mL.

Routine endomyocardial biopsy conducted 9 days post-transplantation revealed a grade 2R acute graft rejection with no humoral rejection (pAMR0), according to the International Society for Heart and Lung Transplantation score. Heart transplantation was assessed by echocardiography, presenting a left ventricular hypertrophy with a normal ejection fraction. Cyclosporin was switched to tacrolimus and IV methylprednisolone (10 mg/kg/d) was administered for 3 consecutive days, followed by an increase in oral methylprednisolone doses. Two weeks later, persistent signs of grade 2R acute rejection with no humoral rejection (pAMR0) were noted on endomyocardial biopsy, requiring a new treatment with IV methylprednisolone (10 mg/kg/d) for 3 consecutive days. Control endomyocardial biopsies over time showed improvement, followed by resolution of signs of acute rejection.

A PET-scan performed during acute rejection treatment showed favorable changes in spondylodiscitis and lymph nodes. Caspofungin dosage was lowered to 70 mg/day 10 weeks after transplantation, relayed by oral fluconazole 3 months after transplantation.

A PET-scan 5 months after transplantation showed complete resolution of all signs of infection. Administering of fluconazole was then stopped.

## 3. Discussion

This is a unique case of cardiac transplantation due to fungal endocarditis. Treatment was only guided by expert opinion. Our patient did not respond to the combination of standard-dose anidulafungin and surgical valve replacement, resulting in our decision to administer higher doses of a different echinocandin, based on the 2016 Infectious Diseases Society of America guidelines’ recommendation of low-quality evidence [4]. We switched to caspofungin, which was not only the first echinocandin approved for clinical use but also the most frequently used in the setting of *Candida* endocarditis [5].

Echinocandins exert their killing effect in a concentration-dependent fashion. Treatment efficacy is achieved by optimizing Cmax/MIC and AUC/MIC, which differ according to the *Candida* species [6]. Higher-than-usual doses of echinocandins are thought to be more effective in difficult-to-treat forms of candidiasis, including endocarditis [7]. Higher doses of caspofungin (150 mg per day) are usually well tolerated, most commonly observed side effects being phlebitis and elevated liver enzymes [8].

The choice of an echinocandin over fluconazole was furthermore justified by the need for better anti-biofilm activity. It has been demonstrated that fluconazole along with voriconazole and posaconazole have no activity on *C. albicans* biofilms. A basic component of the fungal cell wall is the ß-1,3-D-glucan. Since a fungal biofilm’s composition was demonstrated to include cell wall polysaccharides, it was hypothesized that as echinocandins inhibit the ß-1,3-D-glucan, they should have activity against fungal biofilms [5]. Then, in another study comparing the in vitro activity against *Candida* species in biofilms, echinocandins were favored over the combination of amphotericin and azoles [9].

In this case report, there was an indication for heart transplantation as no other surgical therapeutic option was possible, as recommended in the 2023 European Society of Cardiology guidelines [2]. One case report of a native tricuspid valve endocarditis due to *Candida krusei* [10] and another one of a mitral valve *Candida albicans* endocarditis [11] were successfully treated with heart transplantation. Other case reports of bacterial endocarditis treated successfully with heart transplantation were also described in 4 reviews by Guerrero et al. [12], Murphy et al. [13], Givone et al. [14], and Tattevin et al. [11]. In addition to the cases already reported in those studies, an updated review of the literature up to February 2024 presented in Table 1 shows 3 more cases of infective bacterial endocarditis treated by salvage heart transplantation. All cases of fungal endocarditis treated with heart transplantation in the literature are presented in Table 2.

Several multidisciplinary team discussions were needed before deciding to list our patient for a heart transplantation. Arguments in favor of transplantation were that the infection appeared to be only locally uncontrolled (no fever, negative blood cultures, no evidence of new sites of infection, and a Charlson Comorbidity Index of 3 with 77% estimated 10-year survival) and good clinical status of the patient. Arguments against transplantation included the relative contraindication of an active infection, the lack of similar reported cases, shortage of organ donors, and the plan to administer less intensive IS treatment than usual. It should be noted that immunosuppression is also a risk factor when acquiring new fungal and non-fungal infections. Heart transplantation in the case of endocarditis remains limited due to the risk of infection relapse, graft lost, and death after transplantation.

To the best of our knowledge, this is the first case of *Candida tropicalis* prosthetic valve endocarditis, and the third case of fungal infective endocarditis, to be successfully treated with heart transplantation.

To the date of writing this article our patient is alive, doing well and living at home.

## Figures and Tables

**Table 1:** Case reports of bacterial endocarditis treated with heart transplantation (complement to Guerrero et al. [12], Murphy et al. [13], Givone et al. [14] and Tattevin et al. [11]). Abbreviations: M = male; F = female.

Report	Sex & Age	Comorbidities	Valve(s) Involved	Responsible Microorganism	Medical Treatment & Duration	Timing of Transplantation	Outcome
Rzucidło-Resil et al., 2022. [15]	F; 39	severe mitral regurgitation due to myxoid valve disease	Mitral and aortic	Salmonella enterica ssp	Ceftriaxone and trimethoprim/sulfamethoxazole	Several months after the diagnosis (unspecified)	Alive 2 years after transplantation
Van’t Veer et al., 2020. [16]	M; 33	congenital corrected transposition of the great arteries, congenital atrioventricular block, tricuspid valve replacement, and progressive heart failure	Tricuspid	Coxiella brunetii	Doxycycline and hydroxychloroquine, then doxycycline and rifadine, then doxycycline and moxifloxacine, then doxycycline alone	Two years after diagnosis	Alive 5 years after transplantation
Beliaev et al., 2021. [17]	M; 59	rhumatic heart disease, 7 cardiac operations including 6 redo prosthetic heart valve replacements for *C. acnes* endocarditis	Mitral and aortic, and left atrium	Cutibacterium acnes	Lifelong penicillin prophylaxis after transplantation.	Six years after the last prosthetic valve replacement	Alive 11 months after transplantation

**Table 2:** Case reports of fungal endocarditis treated with heart transplantation in the literature. Abbreviations: M = male; F = female; UK = unknown.

Report	Sex & Age	Comorbidities	Valve(s) Involved	Responsible Microorganism	Medical Treatment & Duration	Timing of Transplantation	Outcome
Checchia et al., 2016. [10]	M; 56	Advanced heart failure, diabetes mellitus, arterial hypertension, dyslipidemia, coronary artery disease	Tricuspid	*Candida krusei*	Amphotericin B 4 weeks, switched to anidulafungin for 6 weeks	Four weeks after initiation of amphotericin B	Alive
Tattevin et al., 2023. [11]	UK; 50–60	Unspecified	Mitral	*Candida albicans*	Unspecified	One hundred and sixty-one days after onset of infective endocarditis	Alive
Our case report	M; 63	hemochromatosis, arterial hypertension, hypercholesterolemia, malignant melanoma, chronic kidney disease	Mitral	*Candida tropicalis*	Anidulafungin for 6 weeks, caspofungin for 12 weeks, then fluconazole for 8 weeks, combined then with caspofungin for 16 weeks, then caspofungin alone for 14 weeks, then fluconazole for 8 weeks	Ten months after onset of infective *Candida* endocarditis	Alive

## Data Availability

The original contributions presented in this study are included in the article. Further inquiries can be directed to the corresponding author.
